# A design strategy to match the band gap of periodic and aperiodic metamaterials

**DOI:** 10.1038/s41598-020-73299-3

**Published:** 2020-10-02

**Authors:** Luca D’Alessandro, Anastasiia O. Krushynska, Raffaele Ardito, Nicola M. Pugno, Alberto Corigliano

**Affiliations:** 1 Department of Civil and Environmental Engineering, Politecnico di Milano, Milan, 20133 Italy; 2 Engineering and Technology Institute Groningen, Department of Science and Engineering, University of Groningen, Groningen, 9747AG The Netherlands; 3 Laboratory of Bio-inspired, Bionic, Nano, Meta Materials and Mechanics, Department of Civil, Environmental and Mechanical Engineering, University of Trento, Via Mesiano, 77, 38123 Trento, Italy; 4 School of Engineering and Materials Science, Queen Mary University of London, Mile End Road, London, E1 4NS UK

**Keywords:** Engineering, Materials for devices

## Abstract

The focus of this paper is on elastic metamaterials characterised by the presence of wide sub-wavelength band gap. In most cases, such mechanical property is strictly connected to the periodic repetition of the unit cell. Nonetheless, the strict periodicity requirement could represent a drawback. In this paper, we present a design strategy for aperiodic elastic metamaterials in order to achieve the same performances as for the periodic counterparts. This is done by exploiting the concept of separation of modes for different building blocks, arranged in aperiodic fashion. A theoretical explanation is provided, as well as numerical simulations; the concept is validated by means of a set of experimental tests on prototypes that are realized via additive manufacturing.

## Introduction

Phononic crystals^1,2^ and acoustic metamaterials^3^ with rationally designed architectures can manipulate elastic waves in unprecedented ways. Their structure is responsible for fascinating mechanical and dynamic properties surpassing those of their ingredients, such as e.g. negative effective mass and stiffness^3,4^, negative or extreme acoustic indices^5^, generation of frequency band gaps^1,2^, negative group velocity^6^, one-way and/or scattering-free wave propagation^7^, and others. Despite theoretically unlimited possibilities for design, most researchers focused on periodic configurations characterized by coherent dynamic response, which are easier to study due to the reduction of analysis to a representative building block^3,8,9^. However, a strict periodicity can be unwanted or undesirable from the manufacturing point of view and even can cause spurious effects, such as wave amplification in attenuating materials^3,10^, vibration localization at defects^11,12^, or band-gap suppression in deformed configurations^13^. Furthermore, periodicity appears to be restrictive in realizing more advanced functionalities, e.g. multi-functionality^14^, shape morphing^15^, programmability^16^ or spatially textured response^17^. Recently, a combinatorial strategy has been developed to design frustration-free metamaterials with specified quasi-static mechanical response^17^. In dynamic cases, it has been shown that by displacing identical resonators from periodic arrengement or by using disordered resonators, one can eliminate wave amplification^18^. Other researchers have analyzed the influence of disorder on the geometry of internal resonators and noticed that it may have certain advantages^19–21^.

The presence of abrupt geometrical changes in adjacent cells strongly affects the propagation of low-frequency elastic waves, with the possible consequence of uncontrolled band gap limits or no band gap at all. So far, the break of periodicity has been reported only for acoustic metamaterials with sub-wavelength features and locally resonant band gaps^18,22^. The width of these band gaps is, however, intrinsically limited. Phononic crystals, in contrast, can generate wide band gaps in strictly periodic configurations. In this paper, we demonstrate that the periodicity is not necessary for low-frequency, ultra-wide bandgap, provided that a suitable design strategy is adopted. We implement this strategy by combining cubic blocks originating from different periodic metamaterials, which are based on the mode separation functionality^23^. The latter feature enables to generate ultra-wide band gaps since the dispersion diagram is dominated by low-frequency global modes, at the beginning of band gap, whereas the second pass band is characterized by local modes, for which the wave transmission is strongly attenuated. The filtering behavior of such a class of metamaterials has been shown in previous papers in the case of periodic repetition of the building block. In this paper, we prove that the band gaps are preserved despite geometric discontinuities between the unit cells, if each block is endowed with separation of modes and some global mass and stiffness parameters are preserved. By adopting such a strategy, we can obtain an aperiodic metamaterial endowed with similar band gap features as the periodic counterparts, in spite of the presence of essential geometric differences between the building blocks. This feature substantially extends the space of structural parameters for the design of elastic metamaterials with wide low-frequency band gaps. We provide a theoretical explanation of this fact by means of a simple 1D analytical model, that is compared to the outcomes of 3D numerical simulations. Finally, we realize a sample of an aperiodic meta-structure by means of 3D-printing technique and experimentally prove its broadband wave attenuation ability.

## Design strategy

Our design approach of aperiodic metamaterials relies on the mode separation concept^23^. This concept implies a specific configuration of the unit cell of a periodic metamaterial enabling the separation of vibrational energy between different structural components. For example, a meta-structure formed by periodic alternations of heavy spherical masses and slender ligaments is characterized by passbands with vibration modes confined either in the masses (*global* modes) or in the ligaments (*local* modes). Previous works^23,24^ have shown that the global modes, characterized by the oscillation of the heavy spherical masses connected by the elastic ligaments, is related to the band gap opening, in the low frequency regime. The band gap is closed in correspondence of the local modes, characterized by the vibration of the elastic ligaments themselves, without involving the displacement of the heavy masses. As a consequence, the local modes excite a very small mass (a portion of the mass of the ligament) and the corresponding eigenfrequency is by far higher than the global one. The mode separation results in activation of extremely wide band gaps, as recently shown for several metamaterial configurations^23–26^.

**Figure 1:**
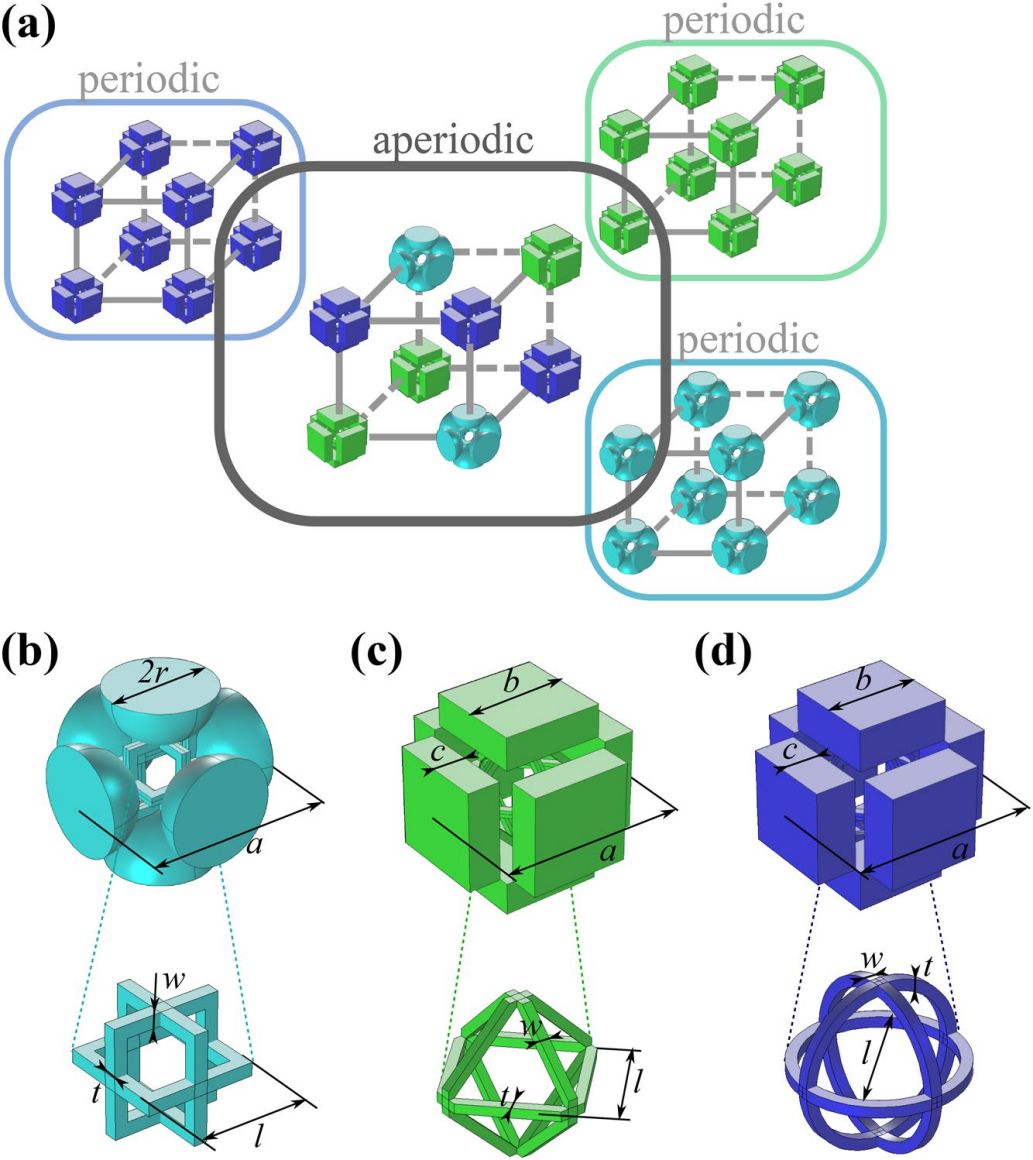
Design strategy and analyzed geometries. (**a**) The composition of periodic and aperiodic metamaterials. (**b**–**d**) Building blocks supporting the mode separation concept: (**b**) “Quad”, (**c**) “Rhomb”, (**d**) “Circle”. The figure has been drawn by the Authors by means of Comsol Multiphysics and Inkscape.

To compose an aperiodic meta-structure, we assemble cubic building blocks into a cubic lattice. The building blocks are represented by unit cells of periodic metamaterials (Fig. 1a). The first unit cell consists of a cubic frame with attached spherical masses and is referred to as “Quad” (Fig. 1b)^23^. Similarly, the second unit cell—“Rhomb”—consists of ligaments arranged in a rhomb-like frame and rectangular cuboid masses (Fig. 1c). The third unit cell is formed by three mutually orthogonal thin rings joining rectangular cuboid blocks and is called “Circle” (Fig. 1d).

The geometric compatibility in an aperiodic metamaterial dictates identical external dimensions for the building blocks. We assign the unit-cell size ${a=5}$ cm that allows one to open a low-frequency band gap in periodic configurations around 2 kHz^23,24^. A band gap in the aperiodic metamaterial can be generated if each periodic configuration has a band gap at the same frequencies. This can be achieved by assigning identical masses to the bulky elements and ensuring comparable effective stiffnesses of the ligaments.

To satisfy these requirements, we assume that each building block is made of identical mono-material. This is Nylon PA12 with Young’s modulus ${E=1.586}$ GPa, Poisson’s ratio ${\nu =0.4}$, and mass density ${\rho =10^3}$ kg/$\hbox {m}^3$^23^. The material choice is governed by the manufacturing (3D printing) technique and the verified procedure of the foreseen experimental tests^23,24^. The spherical or cuboid bulky elements have approximately identical masses, if the radius of the sphere for the “Quad” geometry is ${R=14.9}$ mm, and the width (equal to the height) and half-thickness of the cuboid are ${b=27.2}$ mm, ${c=9}$ mm and $b=25.1$ mm, ${c=11}$ mm, for the “Rhomb” and “Circle” configurations, respectively. Matching effective stiffnesses of connecting frames can be obtained by tuning the geometric parameters of the ligaments. This is done analytically by analyzing equivalent diatomic mass-spring chains, as described in “Interpretation of the physical mechanism via 1D model”.

## Interpretation of the physical mechanism via 1D model

### Description of the model

The design strategy relies on the mechanical explanation of the low-frequency ultra-wide bandgap. As suggested in previous papers^23,24,27^, and following the hints provided in related works^28^ and^29^, the proposed class of metamaterials behaves like a spring-mass chain with an additional resonating element: the interplay between the low frequency modes of the main chain and the high frequency modes of the resonant element leads to the low-frequency, ultra-wide bandgap. In the present work, we propose a new simplified model that is able to describe such a behaviour, catching properly the position and width of the first band gap.

**Figure 2:**
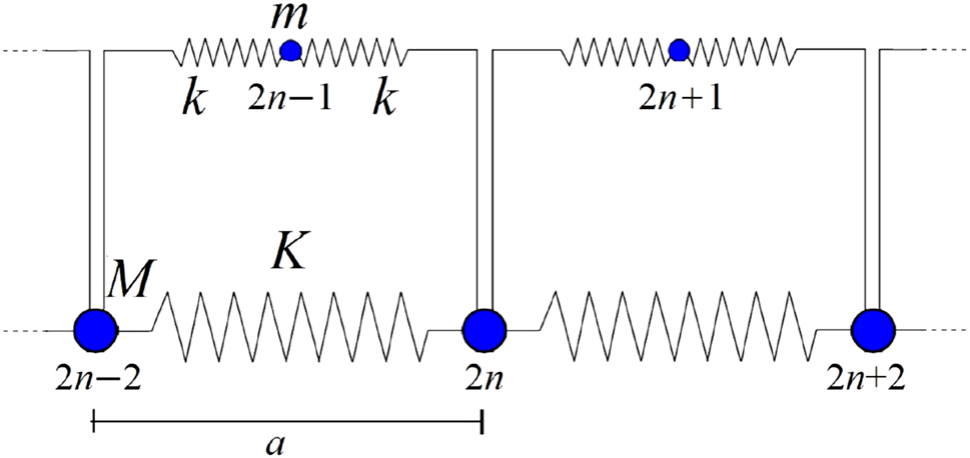
Modes separation mono-dimensional spring-mass chain model.

The proposed model is reported in Fig. 2. The main spring-mass chain is represented by masses *M* connected to one another through the equivalent stiffness given by springs *K* and *k*. The masses *M* physically correspond to the prismatic or spherical elements in the proposed layouts; the connecting stiffness is given by the elastic ligaments (square, circular or rhombic frames). This indicates that the band gap is activated by a mechanism, the *global* mode, that is typically used to explain the behavior of phononic crystals. On the other hand, the flatness of the dispersion bands around the band gap boundaries suggest the presence of a locally resonant mechanism, the *local* mode. The interplay between global and local modes is represented by the masses *m*, which are connected to the main chain through the springs *k*. The parameters *m* and *k* physically correspond to the resonating mass and the modal stiffness for the local modes of the ligaments.

With reference to the model in Fig. 2, the equations of motion are:

1 $$\begin{aligned} M\ddot{u}_{2n}= & {} K\,u_{2n-2}+k\,u_{2n-1}-(2K+2k)\,u_{2n}+k\,u_{2n+1}+K\,u_{2n+2} \end{aligned}$$

2 $$\begin{aligned} m\ddot{u}_{2n+1}= & {} k\,u_{2n}-2k\,u_{2n+1}+k\,u_{2n+2} \end{aligned}$$

A harmonic solution is assumed:

3 $$\begin{aligned} {u}_{2n}= & {} B\,e^{i(\omega t-2nx)} \end{aligned}$$

4 $$\begin{aligned} {u}_{2n+1}= & {} b\,e^{i(\omega t-(2n+1)x)} \end{aligned}$$

where the dimensionless parameter *x* is defined on the basis of the wavelength $\lambda$ and the cell length *a*:

5 $$\begin{aligned} x=\frac{2\pi }{\lambda }\,\frac{a}{2} \end{aligned}$$

By computing the time derivatives, one obtains:

6 $$\begin{aligned} \ddot{u}_{2n}= & {} -B\,\omega ^2\,e^{i(\omega t-2nx)} \end{aligned}$$

7 $$\begin{aligned} \ddot{u}_{2n+1}= & {} -b\,\omega ^2\,e^{i(\omega t-(2n+1)x)} \end{aligned}$$

After substitution in Eqs. (1) and (2), the following system of equations is obtained:

8 $$\begin{aligned} \begin{bmatrix} M\,\omega ^2 - (2K+2k)+(2K\,cos{2x}) &{} 2k\,cosx \\ 2k\,cosx &{} (m\omega ^2-2k) \end{bmatrix} \, \begin{bmatrix} B\\ b \end{bmatrix} = 0 \end{aligned}$$

The non-trivial solution of the system is obtained by setting to zero the determinant of the matrix, which means:

9 $$\begin{aligned} cos^2{x}=-\frac{(\omega ^2-\Omega _{loc}^2)(\omega ^2-2\Omega _{glo}^2)}{4\, \frac{K}{M} (\omega ^2-\alpha ^2\,\Omega _{loc}^2)} \end{aligned}$$

having defined:

10 $$\begin{aligned} \Omega _{loc}^2= & {} 2\frac{k}{m} \end{aligned}$$

11 $$\begin{aligned} \Omega _{glo}^2= & {} 2\frac{K+\frac{k}{2}}{M} \end{aligned}$$

12 $$\begin{aligned} \alpha ^2= & {} 1+\frac{k}{2K}\ge 1 \end{aligned}$$

$\Omega _{loc}$ is the angular frequency of the local mode (oscillation of the mass *m* connected to two springs *k*), whereas $\Omega _{glo}$ is connected to the global mode.

In order to obtain real-valued wave vectors, the following set of inequalities should be fulfilled:

13 $$\begin{aligned} 0\le cos^2{x}\le 1 \end{aligned}$$

which means:

14 $$\begin{aligned} {\left\{ \begin{array}{ll} \frac{\omega ^2-\Omega _{loc}^2(1+ \frac{m}{M})}{(\omega ^2-\alpha ^2\,\Omega _{loc}^2)}\ge 0 \\ \\ \frac{(\omega ^2-\Omega _{loc}^2)(\omega ^2-2\Omega _{glo}^2)}{(\omega ^2-\alpha ^2\,\Omega _{loc}^2)}\le 0 \end{array}\right. } \end{aligned}$$

For the structures where *modes separation* holds, the frequency of the *global* mode is by far smaller than the *local* one: as a consequence, it is possible to assume that $\sqrt{2}\Omega _{glo}<\Omega _{loc}$. Under such an assumption, the solution of the inequalities (14) yields the following passbands:

15 $$\begin{aligned} 0\le & {} \omega ^2 \le 2\Omega _{glo}^2 \end{aligned}$$

16 $$\begin{aligned} \Omega _{loc}^2\le & {} \omega ^2 \le \Omega _{loc}^2\left( 1+ \frac{m}{M}\right) \end{aligned}$$

It is now interesting to study the eigenmodes associated to the limits of these bands. The first passbands start for $\omega ^2=0$, that is clearly associated with the rigid mode of the chain (i.e. $B=b=1$). The passband ends for $\omega ^2=2\Omega _{glo}^2$, that entails $cos^2{x}=0$, on the basis of Eq. (9), and $cos{2x}=-1$. After some algebraic manipulation, the governing system becomes:

17 $$\begin{aligned} \begin{bmatrix} 0 &{} 0\\ 0 &{} 4m\,\frac{K+k/2}{M}-2k \end{bmatrix} \, \begin{bmatrix} B \\ b \end{bmatrix} = 0 \end{aligned}$$

The mode which defines the opening of the first band gap is therefore: $B=1$, $b=0$, which is associated to *global* motion.

The lower limit of the second pass band is now considered, $\omega ^2=\Omega _{loc}^2$. As before, $cos^2{x}=0$ and $cos{2x}=-1$, but the governing system is now represented by:

18 $$\begin{aligned} \begin{bmatrix} 2M\frac{k}{m}-4K-2k &{} 0 \\ 0 &{} 0 \end{bmatrix} \, \begin{bmatrix} B\\ b \end{bmatrix} = 0 \end{aligned}$$

Therefore, the mode which defines the closing of the first band gap is: $B=0$, $b=1$, which is associated to *local* motion. It is worth noting that the first band gap is opened and closed on the same symmetry point, unlike what typically happens for locally resonant materials.

Finally, the upper limit of the second passband is $\omega ^2=\Omega _{loc}^2(1+ m/M)$, that entails $cos^2{x}=1$, therefore $x=n\pi$. The associated eigenvector is characterized by the motion of both masses: $B=-\frac{m}{M}b$.

**Figure 3:**
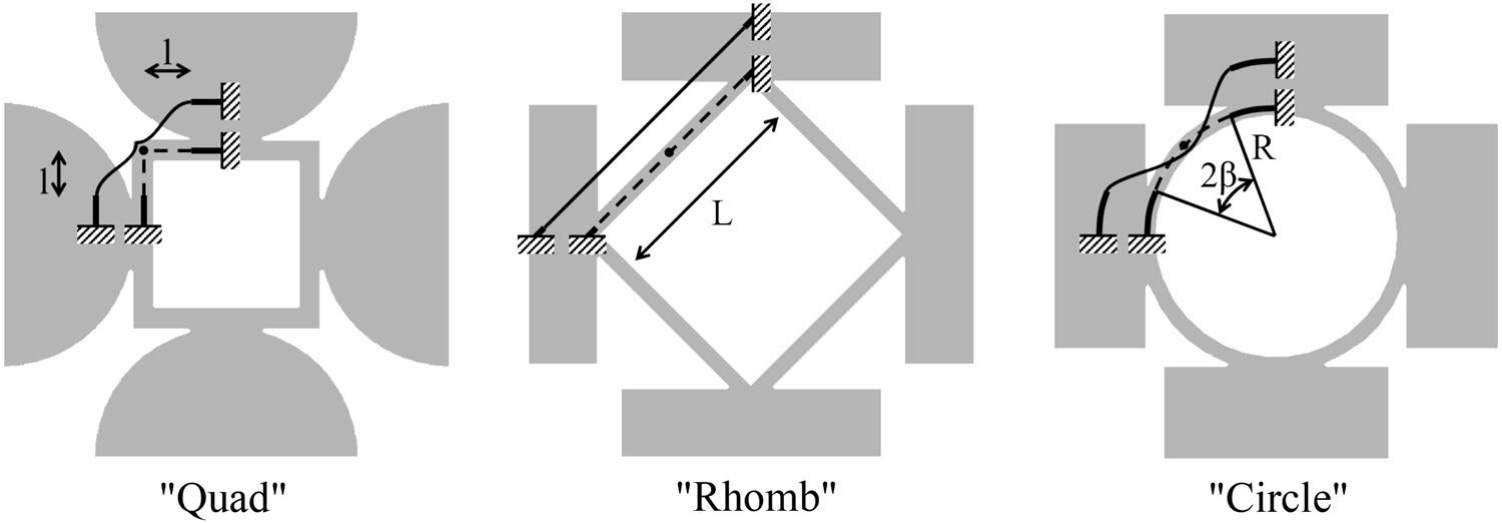
Simplified schemes for the evaluation of *global* and *local* parameters of the three proposed layouts.

For the sake of completeness, we consider the case $\sqrt{2}\Omega _{glo}>\Omega _{loc}$. In that situation, the roles of global and local modes are basically inverted, the first passband is for $0\le \omega ^2 \le \Omega _{loc}^2$ and the second passband involves the movement of the mass *M*. In the limit case $\sqrt{2}\Omega _{glo}=\Omega _{loc}$ mode separation does not exist and there is a unique passband for $0\le \omega ^2 \le (1+m/M) \Omega _{loc}^2$.

### Application to the proposed layouts

The proposed model is now used in order to predict the initial and final frequency of the first band gap for the three cells presented in Fig. 1. The physical correspondence between the lumped parameters and the various components of the cells are explained in the previous section and allow us to compute the mass and stiffness parameters on the basis of the geometric features of the proposed layouts. The interpretative schemes, based on the results achieved in previous works^23,24^, are shown in Fig. 3. The examination of the band gap opening and closing modes suggest that the opening mode is characterized by the simultaneous oscillation of the masses, with the typical deformed shape of the connecting frames depicted in Fig. 3. Consequently, in all the cases the global stiffness coincides with that of the planar frame, which can be computed by studying one quarter of the structure, in view of double symmetry. The global stiffness $(K+k/2)$ is twice the stiffness of the frame, since the masses are connected by another frame, in the orthogonal plane, not shown in Fig. 3. The global mass *M* is given by the spherical or prismatic items. For what concern the local parameters, the examination of previous results suggests that the local modes are given by intrinsic oscillation of the connecting elements. Therefore, the local parameters are computed by considering the out-of-plane oscillation of the single branches of the frame. The local mass *m* is equal to the modal mass of the frame models shown in Fig. 3 and it is concentrated on the mid point.

For the “Quad” case, the global stiffness can be computed by studying the corner frame via the Timoshenko beam model. By assuming the deformation mode depicted in Fig. 3, one finds:

19 $$\begin{aligned} K+\frac{k}{2}=2 \frac{1}{\frac{l^3}{12EI}+\frac{l}{GA^*}+\frac{l}{EA}} \end{aligned}$$

where $$E=1.586$$ GPa and $$G=566$$ GPa are the longitudinal and tangential moduli of the material; $$l=4.689$$ mm is the free length of the beams (the rigid parts are evidenced by thick solid lines in Fig. 3); $$I=wt^3/12=$$ 3.808 $$\hbox {mm}^4$$ is the moment of inertia of the cross-section; $$A=tw=$$ 6.760 $$\hbox {mm}^2$$ is the area of the cross-section; $$A^*=tw/1.2=5.633$$$$\hbox {mm}^2$$ is the reduced area for shear effects. The global mass is given by:

20 $$\begin{aligned} M= \rho \frac{4}{3} \pi r^3 \end{aligned}$$

where $\rho =1000$ kg/$\hbox {m}^3$ is the density of the material; $r=14.9$ mm is the radius of the sphere. The local stiffness is given by the out-of-plane stiffness of each beam that compose the corner frame. The application of the Timoshenko model, account taken of the torsional effect, yields:

21 $$\begin{aligned} k=\frac{1}{\frac{l^3}{12EI} \frac{1+4\frac{EI}{GJ}}{1+\frac{EI}{GJ}}+\frac{l}{GA^*}} \end{aligned}$$

where $$J=0.141tw^3=6.443$$$$\hbox {mm}^4$$ is the torsional inertia of the cross-section. By studying the out-of-plane mode, one finds that the lumped mass can be estimated as the overall mass of the deformable parts of the corner frame:

22 $$\begin{aligned} m= 2 \rho wtl \end{aligned}$$

If one considers the “Rhomb” layout, the global stiffness can be easily computed on the basis of the axial stiffness of the beam:

23 $$\begin{aligned} K+\frac{k}{2}=2 \frac{EA}{L} \end{aligned}$$

where $$L=21.45$$ mm is the free length of the beam; $$A=tw=4$$$$\hbox {mm}^2$$ is the area of the cross-section. The global mass is:

24 $$\begin{aligned} M= \rho 2cb^2 \end{aligned}$$

For the local mode, one can consider the out-of-plane vibration of a clamped-clamped beam with a concentrated mass in the middle. The stiffness of one half of the beam is exactly the local stiffness:

25 $$\begin{aligned} k=\frac{1}{\frac{(L/2)^3}{12EI}+\frac{(L/2)}{GA^*}} \end{aligned}$$

where $$I=tw^3/12=1.333$$$$\hbox {mm}^4$$ is the moment of inertia of the cross-section; $$A^*=tw/1.2=3.333$$$$\hbox {mm}^2$$ is the reduced area for shear effects. The lumped mass is about one third of the mass of the whole beam:

26 $$\begin{aligned} m= \frac{1}{3} \rho wtL \end{aligned}$$

For the “Circle” case, the global stiffness can be computed on the basis of the simplified theory for curved Timoshenko beams^30^. One obtains:

27 $$\begin{aligned} K+\frac{k}{2}=\frac{2}{\frac{R^3}{EI} \left( \beta +\frac{\sin 2\beta }{2} - \frac{1-\cos 2\beta }{\beta } \right) +\frac{R}{GA^*}\left( \beta - \frac{\sin 2\beta }{2} \right) +\frac{R}{EA}\left( \beta + \frac{\sin 2\beta }{2} \right) } \end{aligned}$$

where $$R=14$$ mm is the free mean radius of the circular beam; $$\beta =$$ 0.4515 rad is the half-opening of the free span; $$I=wt^3/12=1.333$$$$\hbox {mm}^4$$ is the moment of inertia of the cross-section; $$A=tw=4$$$$\hbox {mm}^2$$ is the area of the cross-section; $$A^*=tw/1.2=3.333$$$$\hbox {mm}^2$$ is the reduced area for shear effects. The global mass is:

28 $$\begin{aligned} M= \rho 2cb^2 \end{aligned}$$

For the local mode, the same consideration as for the “Rhomb” case hold.

On the basis of the above considerations, one can easily compute the typical frequency in the dispersion diagram for the different models, namely: the frequency that opens the first band gap $f_{op1}=\sqrt{2} \Omega _{glo} / 2\pi$; the frequency that closes the first band gap $f_{cl1}= \Omega _{loc} / 2\pi$; the frequency that opens the second band gap $f_{op2}=\sqrt{1+ m/M} \, \Omega _{loc} / 2\pi$. The obtained results, for the different layouts, are summarized in Table 1.

**Table 1:** Model parameters considering the simplified schemes in Fig. 3.

	“Quad”	“Rhomb”	“Circle”	
*M*	13.86	13.81	13.86	(g)
*m*	0.0722	0.0286	0.0253	(g)
*K*	499.9	582.2	559.3	(N/mm)
*k*	180.1	18.41	75.18	(N/mm)
$K+k/2$	590.0	591.4	596.8	(N/mm)
$f_{op1}$	2077.0	2082.9	2088.8	(Hz)
$f_{cl1}$	11243	5709.6	12273	(Hz)
$f_{op2}$	11272	5715.5	12284	(Hz)

The three layouts have been designed in order to show the same global mass and global stiffness: that entails that the frequency that opens the first band gap is matched between the three cases. On the other hand, the other frequencies are pretty different.

**Figure 4:**
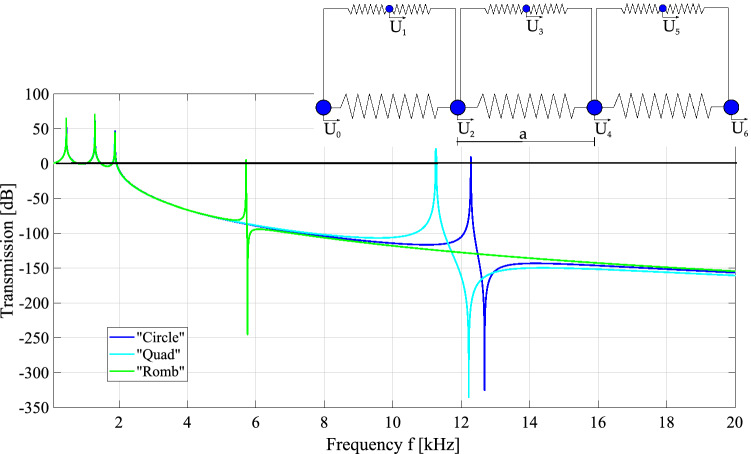
Transmission plots for the simplified schemes presented in Fig. 3.

The one-dimensional model can be also used to carry out transmission analysis, by repeating the unit cells to obtain a finite structure. The equations of motion can be established and solved and eventually the ratio between the amplitude of the signal introduced on the first mass and the amplitude of the signal received on the last mass can be plotted. If one considers three unit cells (see Fig. 1), the transmission curves are reported in Fig. 4. The first bandgap coincides with the zone of negative transmission (i.e. attenuation). The curves are exactly superimposed in the low-frequency pass band; the opening frequency is roughly the same for the three cases, around 2 kHz, and is connected to the global mass-spring chain behavior. The band gap is closed by a sharp transmission peak, suddenly followed by an attenuation peak. This is typical of resonant systems and confirms that the band gap is closed because of the presence of a resonating local mode. Therefore, the examination of the transmission plot confirms the behavior connected to mode separation.

## Numerical and experimental results

### Dispersion analysis

The specification of structural parameters allows performing full-scale finite-element simulations. We start from the dispersion analysis of the periodic configurations performed numerically by means of the Solid Mechanics Module in COMSOL Muliphysics 5.2. We model a single building block of each designed metamaterial and apply the periodic Bloch–Floquet boundary conditions at the three pairs of lateral faces. The geometry is meshed by tetrahedral elements, and the mesh convergence is confirmed. The related eigenfrequency problem is solved for positive values of wave vector at the boundary of the irreducible Brillouin zone (IBZ) for a cubic lattice.

**Figure 5:**
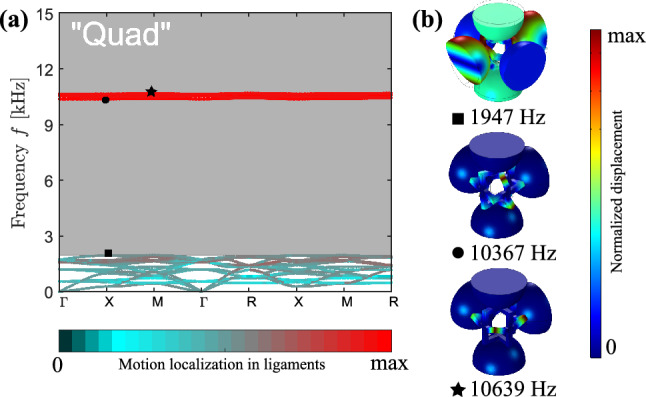
Dispersion analysis. (**a**) Band diagram for the “Quad” periodic metamaterial with the color of the passbands indicating the degree of the motion location in ligaments, as defined in Eq. (29). Band gaps are shaded in gray. (**b**) Vibration patterns at the boundaries of the band gaps (total displacement is normalized to maximum displacement). The figure has been drawn by the Authors by means of Comsol Multiphysics and Inkscape.

**Figure 6:**
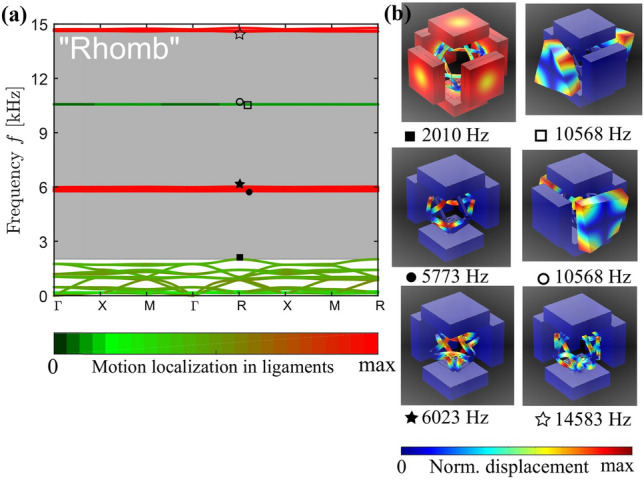
Dispersion analysis. (**a**) Band diagram for the “Rhomb” periodic metamaterial with the color of the passbands indicating the degree of the motion location in ligaments, as defined in Eq. (29). Band gaps are shaded in gray. (**b**) Vibration patterns at the boundaries of the band gaps (total displacement is normalized to maximum displacement). The figure has been drawn by the Authors by means of Comsol Multiphysics and Inkscape.

**Figure 7:**
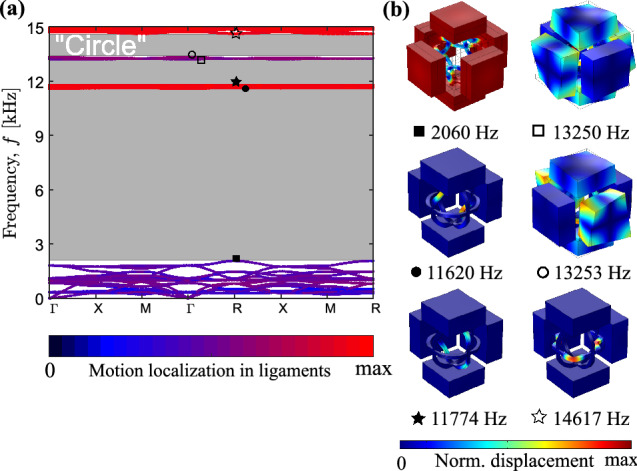
Dispersion analysis. (**a**) Band diagram for the “Circle” periodic metamaterial with the color of the passbands indicating the degree of the motion location in the ligaments, as defined in Eq. (29). Band gaps are shaded in gray. (**b**) Vibration patterns at the boundaries of the band gaps (total displacement is normalized to maximum displacement). The figure has been drawn by the Authors by means of Comsol Multiphysics and Inkscape.

In the framework of the linear elasticity, band diagrams for “Quad”, “Rhomb”, and “Circle” cases are presented in Figs. 5a, 6a and 7a, respectively, with the band gaps indicated in gray. To distinguish between various mode types, the pass bands are coloured according to the amount of the motion localization *p* in ligaments, namely,

29 $$\begin{aligned} p = \frac{\int \limits _{{\hat{V}}}\left( {\left| {\hat{u}}_x\right| ^2+\left| {\hat{u}}_y\right| ^2+\left| {\hat{u}}_z\right| ^2}\right) d{\hat{V}}}{\int \limits _V\left( {\left| u_x\right| ^2+\left| u_y\right| ^2+\left| u_z\right| ^2}\right) d{}V}, \end{aligned}$$

where ${\hat{u}}_x$, ${\hat{u}}_y$, ${\hat{u}}_z$ and ${\hat{V}}$ are the displacements and the volume of the ligaments, while $u_x$, $u_y$, $u_z$ and *V* are the displacements and the total volume of solid parts within a building block. The (almost) zero values of *p*, indicated by dark colors in Figs. 5a, 6a and 7a, correspond to modes localized in the massive elements with almost motionless ligaments. The values ${0.2<p<0.8}$ describe the modes with all parts of the metamaterials involved in motion. These are depicted by colors similar to those of the unit cells in Fig. 1b–d. Finally, the values of ${0.8\le {}p\le {}1}$ correspond to the modes, the motions in which are localized in the ligaments, and are depicted in red.

Note that below the first band gap, the modes are mainly of the mixed, non-localized type (see the first sub-figure in Figs. 5b, 6b, 7b). They can be classified as the *global* modes, as all the unit cell parts are in motion^23^. Instead, above the the first band gap, the modes represented by almost flat curves exhibit a localized character—the *local* modes—with intense motions concentrated either in masses or in ligaments (Figs. 5b, 6b, 7b).

The evaluated band-gap bounds are in a fair agreement with the analytical predictions discussed in “Interpretation of the physical mechanism via 1D model”. For the “Quad” configuration, the calculated frequency ${f_{op1}=1950}$ Hz is 6.5% lower than the predicted value ${f_{op1}^{(a)}=2077}$ Hz. This can be explained by a larger connection area between the ligaments and the spheres, as assumed in the analytical analysis, that decreases the bending stiffness of the frame. For the “Rhomb” unit cell, the numerical lower band-gap bound is only 3.6% smaller than the analytical value (i.e., ${f_{op1}=2010}$ Hz vs. ${f_{op1}^{(a)}=2083}$ Hz) that again results from a larger connection area between the frame and the cuboid blocks. The smallest difference is observed for the “Circle” case, constituting less than 1.5% between ${f_{op1}=2059}$ Hz and ${f_{op1}^{(a)}=2089}$ Hz. For the higher frequency modes, the maximum mismatch between the numerical and analytical results slightly exceeds 8%. Hence, we conclude that the proposed spring-mass models properly capture the dispersion of both *global* and *local* modes of three-dimensional metamaterials supporting the mode separation functionality. This is particularly attractive, taking into account that the dynamics of the designed metamaterials is characterized by complex, essentially three-dimensional motions with non-uniform displacements of different components of the building blocks (see, e.g., the first sub-figure in Figs. 5b, 6b, 7b).

In contrast to the lower band-gap bounds, the corresponding upper bounds are difficult to match due to (1) a large number of imposed geometric constraints (unit-cell dimensions, ligament geometry, limitations originating from the manufacturing and exploitation requirements, e.g. the ligaments should be thick enough to maintain the structural stability) and (2) different deformation mechanisms in the *local* modes governed by different sets of mechanical parameters (see “Interpretation of the physical mechanism via 1D model”). As the normalized gap width (the ratio between a gap width and a mid-gap frequency) for the “Quad”, “Rhomb”, and “Circle” configurations is large (i.e., 137%, 97%, and 140%, respectively), and the upper bounds are formed by localized modes, the aperiodic metamaterial can have the attenuation performance typical for a low-pass filter, when the attenuation starts from the lower band-gap bound and proceeds beyond the upper bound, merging the subsequent band gaps^23^. To verify this, we perform experimental and numerical transmission analysis on finite-size samples.

**Figure 8:**
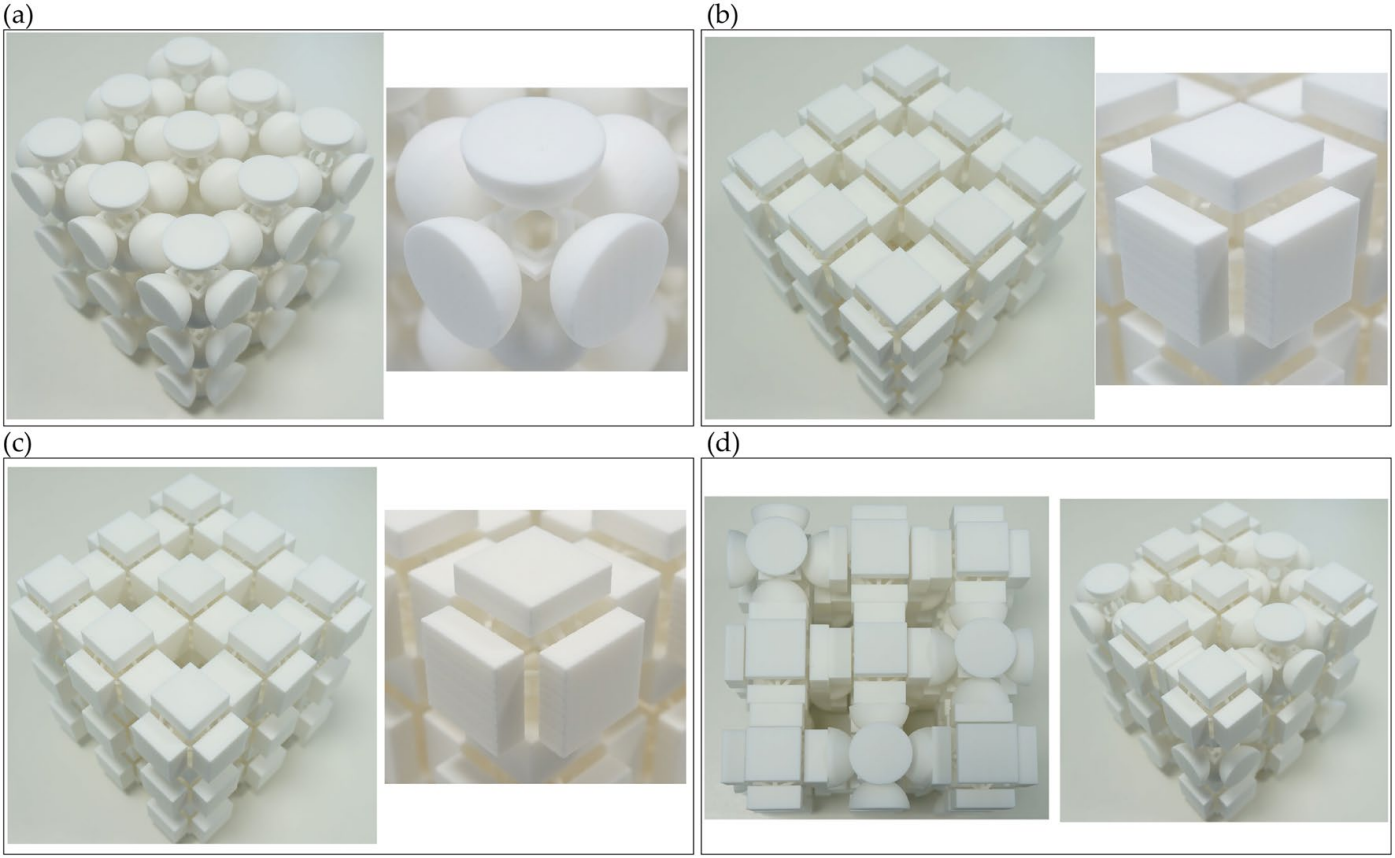
Prototypes of the designed geometries. The periodic samples (**a**) “Quad”, (**b**) “Rhomb”, (**c**) “Circle”, and the “aperiodic” structure (**d**). The figure contains photographs of the prototypes, taken by the Authors.

### Transmission analysis

The dispersion analyses, reported in the previous section, are used to study the three layouts in the hypothesis of infinite periodic repetition. On the other hand, transmission analyses for finite samples can be adopted to study both the periodic and the aperiodic systems and to compare the achieved results, numerically and experimentally. The experiments aim at confirming the filtering behavior of the periodic metamaterials and at proving that the aperiodic metamaterial behaves in the same way as the periodic ones, i.e. that filtering properties are not destroyed by the absence of geometric periodicity. Prototypes with periodic and aperiodic arrangements of ${3\times 3\times 3}$ building blocks are produced by means of the Selective Laser Sintering (SLS) technique from Nylon PA12 with mechanical parameters indicated in “Design strategy” (see Fig. 8). The chosen number of the building blocks is compatible with the rapid manufacturing technique and sufficient to correctly capture the band gap attenuation regimes (for more details see Ref.^27^). As a matter of fact, the finite size of the lattice may affect the dynamic response, but the proposed arrangement is large enough to ensure the correct representation of the lattice behavior. The confirmation will be given by the examination of the linear-elastic transmission plot: the attenuation zones must agree with the band gaps reported in the dispersion diagrams.

**Figure 9:**
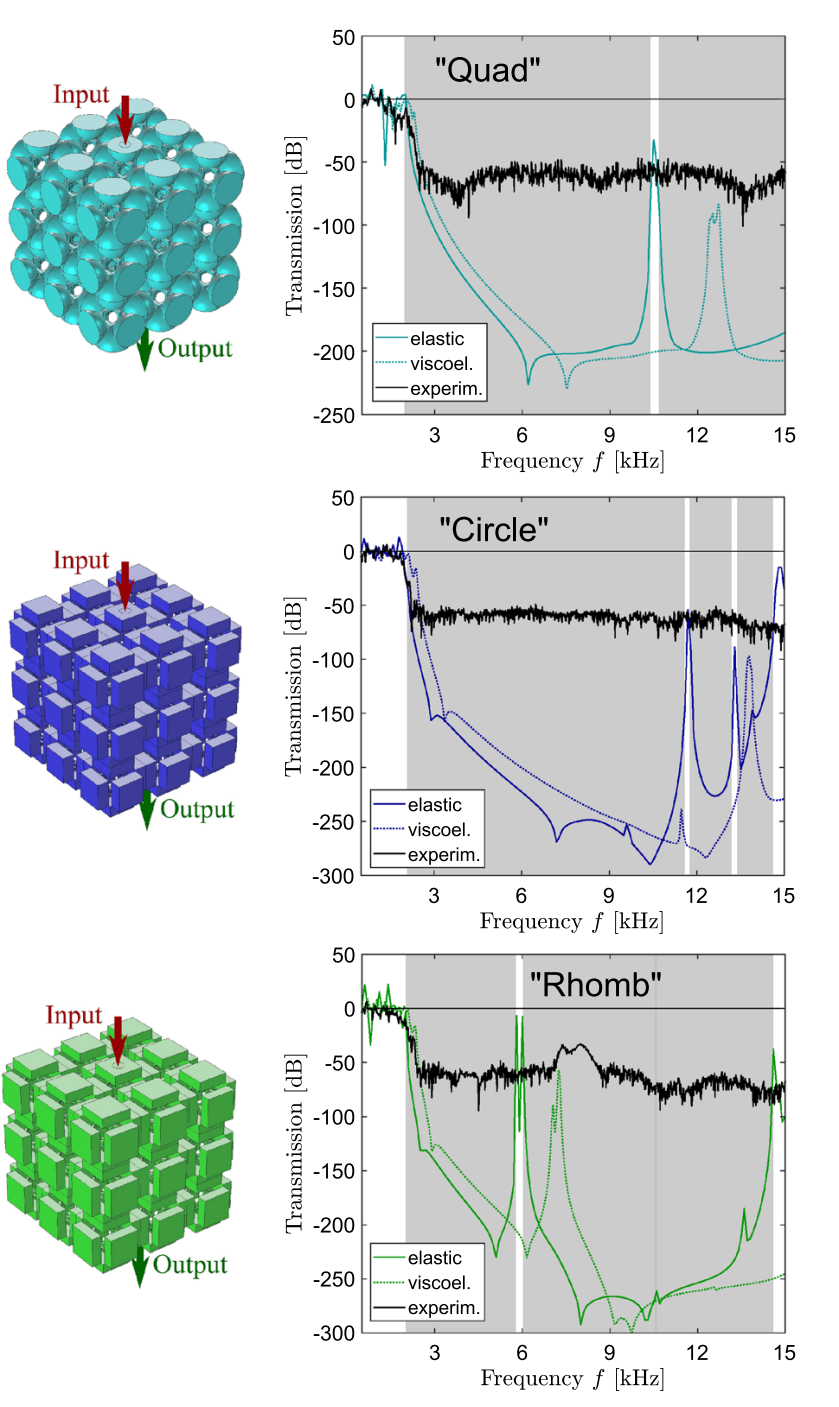
Transmission analysis. Experimental transmission results for the the periodic metamaterial samples (the black solid lines); numerical transmission data for the same configurations for linear elastic material behavior (the colored dashed lines) and viscoelastic behavior (the colored solid lines). The figure has been drawn by the Authors by means of Comsol Multiphysics and Inkscape.

**Figure 10:**
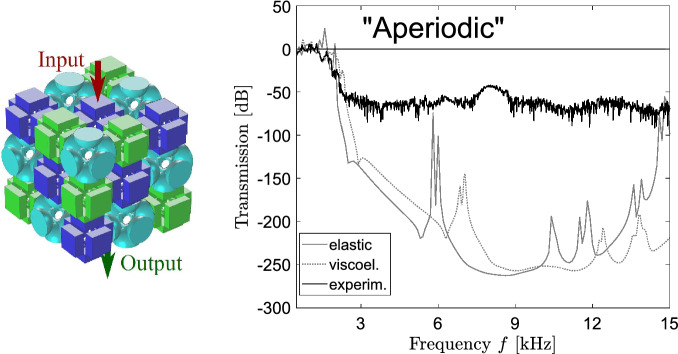
Transmission analysis. Experimental transmission results for the “Aperiodic” metamaterial sample (the black solid line); numerical transmission data for the same configurations for linear elastic material behavior (the colored dashed line) and viscoelastic behavior (the colored solid line). The figure has been drawn by the Authors by means of Comsol Multiphysics and Inkscape.

The transmission spectrum is measured along the ${\Gamma -X}$ direction of the IBZ that allows catching the band-gap frequencies due to the structural symmetry. To this purpose, a prototype is placed on a bubble wrap to isolate it from environmental vibrations. A VibeTribe-Mamba with 20 W power and a frequency range from 40 Hz to 22 kHz is used as actuator. A harmonic excitation is applied on a circular input area (see Figs. 9, 10) at one side of a cubic sample in the orthogonal direction to the surface, while the output signal is detected at the opposite face along the same direction. To sense the input and output signals, two PCB Piezotronics 353 B 15 accelerometers (10 mV/g sensitivity and 70 kHz resonant frequency) are glued to the designated surface areas. The data acquisition chain is completed with an 8-channel PCB 483 C 05 ICP Sensor Signal Conditioner and a NI 9205 module (16-bit resolution). The experimental tests are performed with a 60-s white noise from 0.2 to 15 kHz and by using a rectangular window function. Acquired signals are sampled 51,200 times per second and post-processed by means of Bartlett’s method by dividing them into 200 segments to guarantee a sufficient frequency resolution (i.e. $\delta {}f$ = 3.3 Hz). The transmission is then defined as a ratio between the output to input force amplitudes expressed in dB. Note that the described experimental setup can measure a maximum attenuation up to 75 dB (i.e. 3.75 orders of magnitude).

The measured data are given in Figs. 9 and 10 by the black solid lines together with the numerical curves estimated by using the Solid Mechanics Module of Comsol Multiphysics v5.2. In the simulations, we analyze the same metamaterial configurations as in the experiments and first apply a linear elastic model for the Nylon material. At the unloaded surfaces of the samples, stress-free boundary conditions are applied. The numerical transmission data are shown by the dashed lines in Figs. 9 and 10 and perfectly match the band-gap frequencies predicted by the dispersion analysis, which are highlighed in gray. This confirms the accuracy of the transmission simulations and sufficiency of the chosen number of building blocks to capture the band-gap features. However, one observes certain discrepancies between the calculated and experimental results. First, the experimental band gaps are not aligned with the numerically predicted transmission drops being shifted towards higher frequencies. This is a clear indication of the viscoelastic material behaviour^9,24,27^. To capture viscoelastic effects, we apply a simple standard linear solid model ${{\varvec{\sigma }^{(ve)}}=2(G'+iG''){\varvec{\epsilon }}^{(el)}}$ with $G'=G^{(ve)}\frac{(\omega {}\tau ^{(ve)})^2}{1+(\omega {}\tau ^{(ve)})^2}$ and $G''=G^{(ve)}\frac{\omega {}\tau ^{(ve)}}{1+(\omega {}\tau ^{(ve)})^2}$ for Nylon^27^ (the superscripts (*ve*) and (*el*) refer to viscoelastic and elastic material behavior, respectively).

The coefficients of the model, the relaxation time $\tau ^{(ve)}=$8.95e-4 s and the relaxation shear modulus ${G^{(ve)}= 235}$ MPa, are properly calibrated to catch the attenuation characteristics of the two lowest passbands for the 3D-printed prototypes. In general, the frequency-dependent behavior of 3D printed material is quite difficult to be captured, in view of the fact that the polymerization is uneven in different regions of the prototypes (specifically, in the bigger parts the polymerization is complete on the outer layer, whereas the internal material structure is largely unknown). The simple viscoelastic model has been chosen with the purpose of understanding the frequency shift and the attenuation of the experimental passbands. The viscoelastic transmission simulations are shown by the dashed solid lines in Figs. 9 and 10.

The comparison between experimental data and numerical outcomes is fair. One observes that in the first passband, until about 1.8 kHz, the viscoelastic numerical simulations reproduce correctly the experimental data: to appreciate that fact, the zoomed views of the comparative plots in the range 0.5–3 kHz are reported in the Supplementary Information 1. It is worth noting that, for all the cases, there is a discrepancy between experiments and simulations in the frequency range around 2 kHz: this is possibly due to the insufficient damping, for that specific frequency range, connected to the simple viscoelastic model. At higher frequencies, the simulations predict large attenuation, until 300 dB. Such an attenuation cannot be captured by the experimental apparatus, that is able to measure transmission until − 75 dB because of the intrinsic limitation of the accelerometers. As a matter of fact, the coherence plots (reported in the Supplementary Information) show in general null values beyond 2 kHz, which testifies the noisy nature of the measurement in that frequency regions. The only exception is represented by the case “Rhomb”. After the examination of the coherence plot, it is possible to conclude that the second passband, between 7.5 and 8 kHz, is not an experimental artifact and should be captured by the model. Figure 9, case “Rhomb”, shows that the viscoelastic model is able to capture the frequency shift of the second passband with a reasonable accuracy (error in the frequency peak slightly less than 10%). The attenuation is larger than expected, but not unreasonable. In all the other cases, it is not possible to check the experimental/numerical agreement in the noisy region and we cannot exclude the presence of resonant modes, as predicted by the viscoelastic simulations. Nonetheless, such modes correspond to attenuations larger than 80 dB, i.e. four orders of magnitude.

The experimental results for the periodic arrangements, Fig. 9, confirm the numerical prediction in terms of ultra-wide low-frequency band gap. Moreover, it is possible to notice that higher frequency passbands separating adjacent band gaps almost completely disappear in the experimental and numerical viscoelastic data for the periodic configurations. This can be explained by the localized character of these modes (see Figs. 5, 6, 7) excluding their proper excitation in standard transmission tests. Therefore, the adjacent band gaps are merged and all the designed metamaterial structures act as filters for waves above 2 kHz.

The most important result is referred to the aperiodic sample, see Fig. 10. Indeed, both the numerical and the experimental results confirm that the band gap is present and that the opening frequency coincides with the value that characterizes the three building blocks. This proves that the lack of periodicity does not affect the filtering behaviour. This claim is strengthened by additional analyses, reported in the Supplementary Information 1, for different random arrangements of the aperiodic material: in all the cases, the numerical transmission plots show the same band gaps.

## Conclusions

The aim of this paper is to validate the possibility of realizing aperiodic metamaterials with band gap properties that match the behavior of the periodic cases. The design strategy is based on the assembly of various building blocks, characterised by completely different geometric features. The dispersion spectrum of each building block is characterized by the presence of wide low-frequency band gap. The proposed metamaterial is aperiodic, since the geometry of the unit cell is not periodically repeated, but some basic mechanical features are preserved. As a matter of fact, the behavior of the proposed metamaterials can be interpreted by means of an analytical model, which shows that the opening and closing frequency of the first band gap are determined by *global* and *local* mass/stiffness parameters, respectively. The different building blocks have been designed with the objective of having the same mass and stiffness connected to global mode, so that the opening frequency is the same. On the other hand, the mass and stiffness parameters for the closing frequency are different, as it is quite difficult, in view of the geometric constraint, to match both global and local parameters between the three blocks. The 3D unit cells are thus characterized by different bandgap, with similar opening frequency (maximum difference about 5.6%) and different closing frequency. The aperiodic metamaterial, constituted by the arrangment of the different blocks in a $3\times 3\times 3$ setup, is analyzed by means of numeric and experimental transmission diagrams, that show a wide band gap whose opening frequency is in agreement with the single blocks. The experimental results show also that, in view of the damping connected to viscoelastic behavior of the material, the high frequency passband is strongly attenuated, so that the adjacent band gaps are merged and the metamaterial behaves as a low-pass mechanical filter. This kind of dynamic response is shown in the numeric viscoelastic analyses as well. The achieved results lead us to an important conclusion that we designed the first aperiodic metamaterial with extremely wide low-frequency band gaps, attenuating waves with the efficiency comparable to the periodic counterparts. This paves the way for research developments in various directions. First, the proposed design approach has a potential to reduce the production costs by implementing an alternative manufacturing technique for phononic materials. As discontinuities in the bulky parts do not influence the wave attenuation in a structure, one can produce each unit cell separately, as suggested in the recent literature^31^, instead of a time-consuming 3D-printing of a whole structure. Such parallelization can largely decrease the production time and foster practical applications of the proposed metamaterials. In view of possible industrialization, the presence of defects and of differences between the building blocks cannot be excluded: it would be interesting to investigate to what extent the difference between the band gaps of the unit cells is affecting the presence and the width of the band gap in the aperiodic material. This could be done, for instance, by means of Monte Carlo simulations for a random variation of the mechanical parameters in predefined intervals. Moreover, despite the limitations imposed by the compatibility of the unit cells, the design space of aperiodic metamaterials is huge and not restricted to the configurations supporting the mode separation concept. Finally, the extension of the aperiodic metamaterial concept to the microscopic scale is desirable^32^, in view of the possible application as mechanical or acoustic filter in the field of Micro and Nano Electro-Mechanical Systems (MEMS/NEMS).

## Supplementary information

Supplementary Information 1
