# Supplementary material for: A design strategy to match the band gap of periodic and aperiodic metamaterials
Source: Sci Rep. 2020 Oct 2;10:16403. doi: 10.1038/s41598-020-73299-3 (PMC7532198; doi:10.1038/s41598-020-73299-3)
Supplement: Supplementary file 1 — Supplementary Information 1 [file 41598_2020_73299_MOESM1_ESM.pdf]

A design strategy to match the band gap of periodic and aperiodic metamaterials. Supplementary Information

Luca D'Alessandro[1], Anastasiia O. Krushynska[2], Raffaele Ardito[1], Nicola M. Pugno[3,4], and Alberto Corigliano*[1]

[1]Department of Civil and Environmental Engineering, Politecnico di Milano, Milano 20133, Italy
[2]Engineering and Technology Institute Groningen, Department of Science and Engineering, University of Groningen, Groningen 9747AG, the Netherlands
[3]Laboratory of Bio-inspired, Bionic, Nano, Meta Materials & Mechanics, Department of Civil, Environmental and Mechanical Engineering, University of Trento, Via Mesiano, 77, 38123 Trento, Italy
[4]School of Engineering and Materials Science, Queen Mary University of London, Mile End Road, E1 4NS London, United Kingdom

Coherence plots for the measured data

The coherence γ^2 between the experimental input and output accelerations is computed on the basis of the cross-spectral density S_{io} and the autospectral densities S_{ii} and S_{oo}:

$$\gamma^2(f) = \frac{|S_{io}(f)|^2}{S_{ii}(f)S_{oo}(f)} \tag{1}$$

For linear systems, as the considered metamaterials, the coherence should be ideally equal to unity. Low values of the experimental coherence typically denote the presence of noise. Fig. S1 shows the coherence for the four prototypes, namely the three periodic samples ("Quad", "Circle" and "Rhomb") and the aperiodic one. The coherence is reasonably close to unity only in correspondence of the first passband, i.e. for frequency lower than 2 kHz. Beyond that threshold, the band gap begins and the attenuation attains extreme values, that cannot be captured by the experimental setup in view of the accuracy of the accelerometers. As a consequence, the measurement is dominated by the noise and the coherence is pretty close to zero. The only exception is represented by the case "Rhomb", where the coherence is different than zero (though very small) in the frequency region around 8 kHz. This suggest the fact that the second passband is experimentally detected, even if a rather noisy signal is present.

In order to provide an indication of the region in which the noise overwhelms the measurement, we consider the frequency threshold after which the coherence is lower than 10^{-3}: case "Quad", 2.37 kHz; case "Circle", 2.20 kHz; case "Rhomb", 2.33 kHz; case "Aperiodic", 2.17 kHz. These values are used in the examination of the transmission plot, see Fig. S2 to get better insight in the numerical/experimental comparison.

Numerical-experimental comparison in the low frequency range

The examination of the coherence plots, presented in Fig. S1, confirms that the most reliable results are confined in the first passband, for waves with frequency between 0.5 kHz and 2 kHz. In order to appreciate the accuracy of the numerical models, a zoomed view of the transmission plots in the range 0.5 kHz-3 kHz are presented in Fig. S2. In these plots, the vertical red dashed lines represent the coherence threshold, i.e. the frequency threshold after which the signal-to-noise ratio is no longer acceptable. Moreover, the gray shaded areas represent the band gap as predicted by the dispersion analyses in the linear elastic case.

For the case "Quad", the viscoelastic analysis predicts correctly the experimental behavior around 0.5 kHz. The frequency of the first amplification peak, around 0.82 kHz is slightly overestimated, as it happens for the second peak around 1.15 kHz. In

*Corresponding author, alberto.corigliano@polimi.it

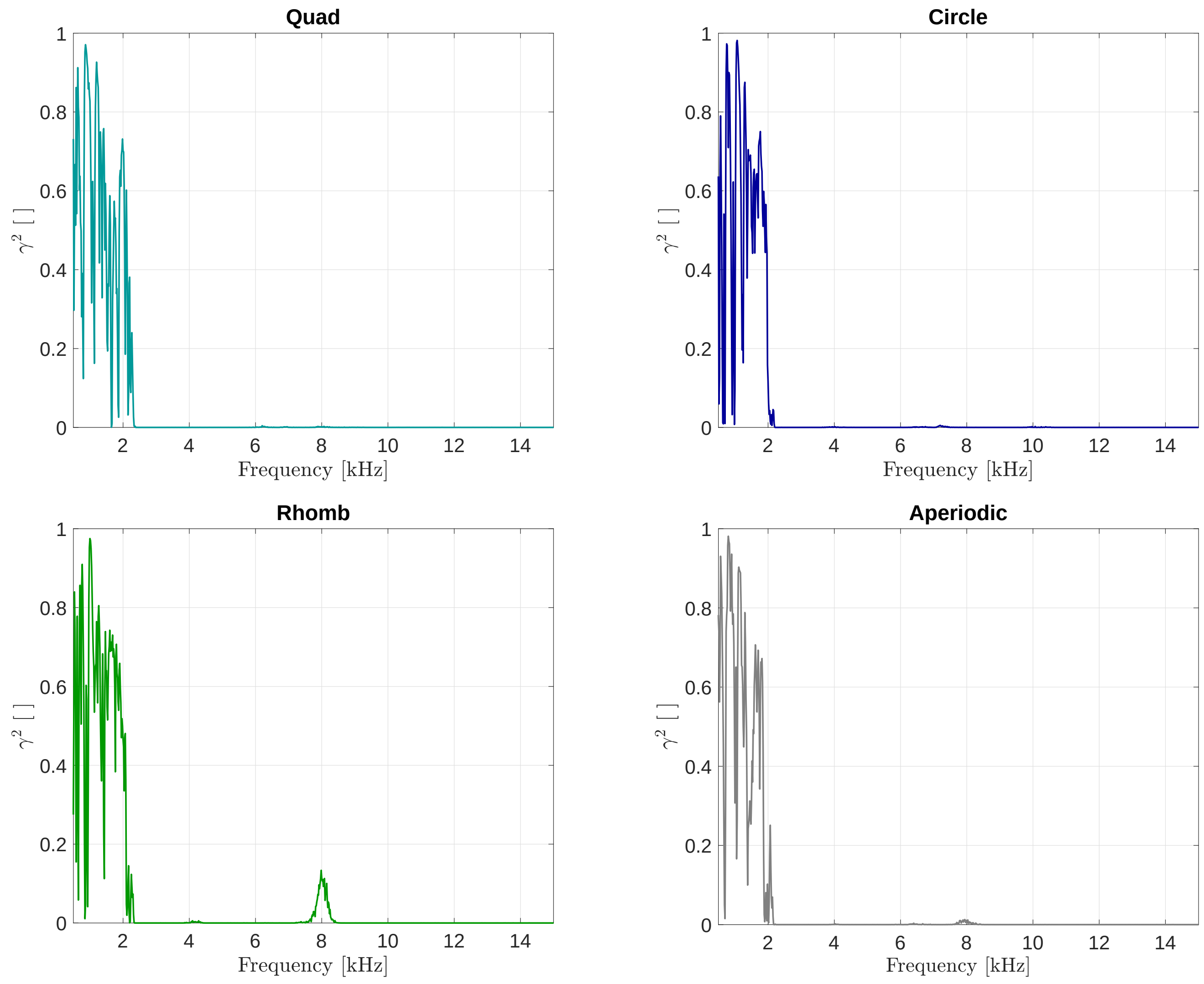

Figure S1. Coherence plot of the input/output measured acceleration for the four tested specimens, namely the three periodic prototypes “Quad”, “Circle” and “Rhomb” and the aperiodic one.

the frequency region around the band gap opening, that is predicted at 1.95 Hz for linear elastic behavior, there is a certain discrepancy in terms of attenuation level: this is due to the fact that the viscoelastic model is very simple, if compared to the complex behavior of the 3D printed material, and that some additional damping would be needed in that frequency range. Nonetheless, the frequency of the small peak at 2.27 kHz, that marks the experimental band gap opening, is correctly simulated by the viscoelastic model. Such a data is reliable, since it lies in the high-coherence region.

In the case “Circle” the discrepancy in the region around the band gap opening is more evident. On the other hand, in this case there is excellent agreement in the initial part of the plot, specifically in correspondence of the first peak (about 0.72 kHz) and of the second peak (about 1.02 kHz). The descent after band gap opening seems too much shifted towards the high frequencies, but this is possibly due to the presence of a numerical peak around 2.37 kHz, that is present in the viscoelastic analysis but it is not visible in the experiments. The discrepancy in this case can be explained by considering the fact that the coherence threshold is pretty close to the elastic band gap opening, so the measurements around 2.37 kHz are dominated by the experimental noise.

The examination of the transmission plots for the case “Rhomb” emphasizes the fact that the viscoelastic analyses are by far better than the elastic ones. Indeed, the elastic analyses shows peaks and troughs with significant amplitude, whereas the experimental transmission in the low frequency regime lies in the range between -10 dB and 6 dB. The viscoelastic transmission is in fair agreement with the experimental data, even though the frequency of the peaks seems to be slightly overestimated. Again, there is a discrepancy around the band gap opening (2.01 kHz for the linear elastic case), that can be ascribed to the lack

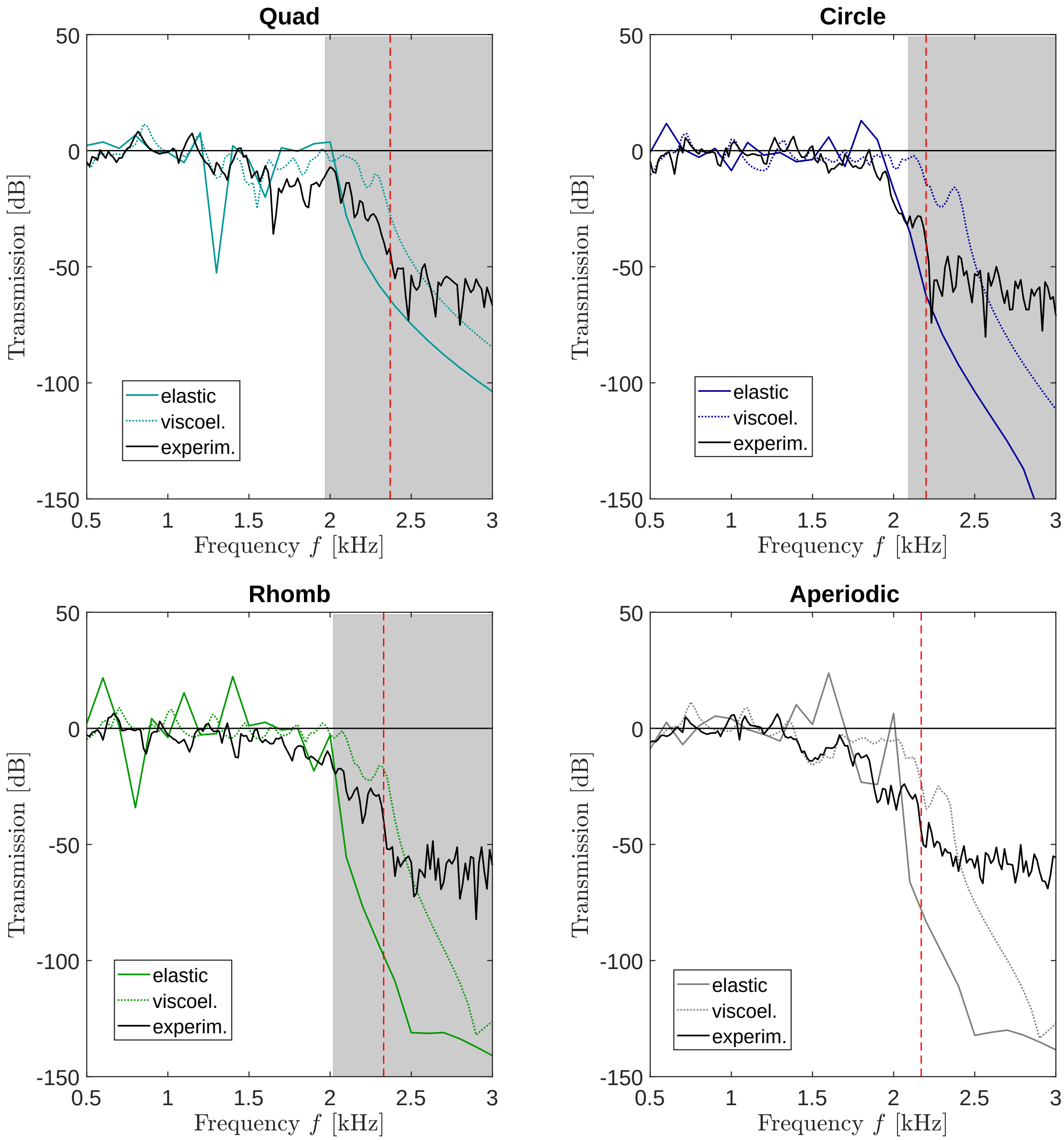

Figure S2. Transmission plots for the four tested specimens, namely the three periodic prototypes “Quad”, “Circle” and “Rhomb” and the aperiodic one. The experimental measurement (solid black lines) are compared to the numerical elastic (solid color lines) and viscoelastic (dashed color lines) analyses. The red dashed line correspond to the coherence threshold, arbitrarily chosen as $\gamma^2 = 10^{-3}$

of damping connected to the viscoelastic model in that frequency regime. The experimental band gap opening is well aligned with the frequency threshold, at 2.33 kHz, and is suitably predicted by the viscoelastic model.

Finally, for the aperiodic metamaterial the match between experimental data and viscoelastic analysis is satisfactory until about 1.8 kHz, with a good prediction of the first and second peaks and an excellent agreement in the trough around 1.48 kHz. The attenuation around 2 kHz is underestimated, that configures a common feature of the viscoelastic model, but, on the other hand, the sudden drop after 2.13 kHz (that represent the band gap opening) is caught with sufficient accuracy. The numerical analyses show a small peak around 2.27 kHz, but that frequency is beyond the coherence threshold.

To conclude, the examination of the numerical and experimental plots in the low frequency regime (from 0.5 kHz to 3 kHz)

shows that the viscoelastic model is able to capture the main features of the elastic wave transmission, even if some additional damping should be introduced in the region around 2 kHz. The comparison is corroborated by the addition of the coherence data, that helps to explain some discrepancy.

Numerical results for different aperiodic arrangements

To support the key claim of this work, we consider other five aperiodic arrangements of the metamaterial building blocks. The aperiodic patterns are created by using uniformly distributed random numbers 1, 2 and 3, referring to "Circle", "Rhomb", and "Quad" building blocks, respectively, and generated by the Matlab function *rand(3,3)*. Each run of the function delivers a 3 by 3 array of the numbers indicating the arrangement of the building blocks in a single layer. The layers can be used to form various aperiodic metamaterial configurations. In two of the analyzed cases, the metamaterials contain 9 building blocks of each type that are arranged as indicated in Table S1.

Table S1. Additional aperiodic arrangements, characterized by the same number of each building block

	Layer 1			Layer 2			Layer 3		
Mix I	3	1	2	2	3	1	3	3	1
	3	2	2	2	1	1	3	2	2
	2	3	2	1	1	3	1	1	3
Mix II	1	2	3	2	3	2	2	2	1
	3	2	1	2	1	1	3	1	2
	1	3	2	3	3	1	1	3	3

The transmission data for these two configurations are given in Figure S3 by the dashed purple and olive curves, respectively. In the other three cases, the building blocks of a single type are dominant. Specifically, we analyzed the arrangements reported in Table S2. As can be seen, the mix 1 has a larger number of the "Circle" building blocks indicated by number 1, and the mixes 2 and 3 are dominated by the "Rhomb" and "Quad" building blocks, respectively. The evaluated transmission results are shown in Figure S3 in blue, green and cyan solid lines. The gray shaded areas indicate the band gaps obtained for the aperiodic arrangement reported in the paper.

The examination of the transmission plots, obtained for a linear elastic constitutive model, confirms that the band gaps are not altered by the different aperiodic arrangements. More specifically, all the cases share the same opening frequency for

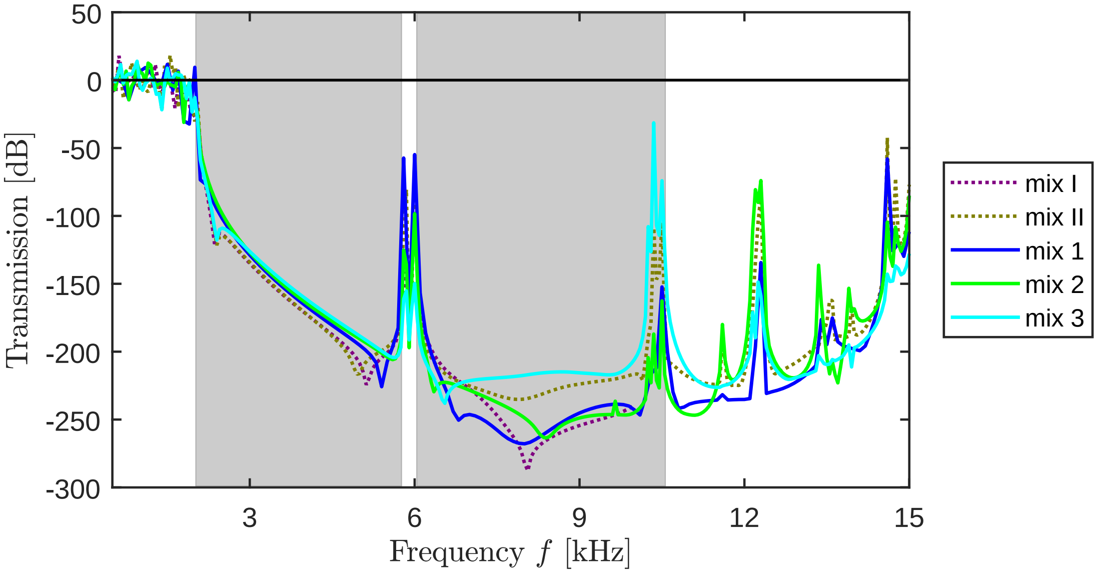

Figure S3. Transmission plots for the different aperiodic arrangements, specified in Table S1 and Table S2. Gray shaded areas represent the first and second band gaps for the arrangement considered in the paper.

the first band gap, around 2 kHz. The pass band around 6 kHz is present for all the cases, with different amplifications. The obtained result supports the statement that the aperiodic metamaterial is endowed with the same filtering properties as the periodic counterparts.

Table S2. Additional aperiodic arrangements, characterized by a dominant building block (boldface number)

	Layer 1			Layer 2			Layer 3		
Mix 1	2	3	**1**	**1**	2	**1**	3	**1**	3
	**1**	2	2	3	**1**	2	2	3	2
	3	**1**	**1**	**1**	2	3	**1**	**1**	3
Mix 2	**2**	1	**2**	1	1	**2**	**2**	1	**2**
	**2**	1	1	3	1	**2**	**2**	3	**2**
	3	1	**2**	**2**	**2**	3	3	3	1
Mix 3	1	2	2	**3**	**3**	**3**	2	1	1
	**3**	**3**	**3**	2	**3**	**3**	1	1	**3**
	**3**	1	**3**	1	**3**	2	**3**	1	**3**
